# Complete post-operative resolution of “*temporary*” end-stage kidney disease secondary to aortic dissection without static renal artery obstruction: a case study

**DOI:** 10.1186/s12882-019-1559-8

**Published:** 2019-10-15

**Authors:** Yoshihiro Mukaiyama, Akira Okada, Yutaro Kawakatsu, Satoshi Akuzawa, Kazuchika Suzuki, Naoyuki Ishigami, Tatsuo Yamamoto

**Affiliations:** 1 Department of Urology, Takashimadaira Chuo General Hospital, 1-73-1 Takashimadaira, Itabashi, Tokyo, 175-0082 Japan; 2 Divison of Nephrology and Endocrinology, The University of Tokyo Graduate School of Medicine, 7-3-1, Hongo, Bunkyo-ku, Tokyo, 113-8655 Japan; 3 Department of Nephrology, Fujieda Municipal General Hospital, 4-1-11 Surugadai, Fujieda, Shizuoka 426-8677 Japan; 4 Department of Cardiovascular Surgery, Fujieda Municipal General Hospital, 4-1-11 Surugadai, Fujieda, Shizuoka 426-8677 Japan

**Keywords:** Aortic dissection, Acute kidney injury, End-stage kidney disease, Surgery, Static obstruction, Dynamic obstruction, Upstream aortic constriction

## Abstract

**Background:**

Acute kidney injury (AKI), which may progress to end-stage kidney disease (ESKD), is a potential complication of aortic dissection. Notably, in all reported ESKD cases secondary to aortic dissection, imaging evidence of static obstruction of the renal arteries always shows either renal artery stenosis or extension of the dissection into the renal arteries.

**Case presentation:**

We present the case of a 58-year-old man with hypertension who was diagnosed with a Stanford type B aortic dissection and treated with medications alone because there were no obvious findings indicative of dissection involving the renal arteries. He had AKI, which unexpectedly progressed to ESKD, without any radiological evidence of direct involvement of the renal arteries. Thus, we failed to attribute the ESKD to the dissection and hesitated to perform any surgical intervention. Nevertheless, the patient’s hormonal levels, fractional excretion values, ankle brachial indices, and Doppler resistive indices seemed to indirectly suggest kidney malperfusion and implied renal artery hypo-perfusion. However, abdominal computed tomography imaging only revealed progressive thrombotic obstruction of the false lumen and compression of the true lumen in the descending thoracic aorta, despite the absence of anatomical blockage of renal artery perfusion. Later, signs of peripheral malperfusion, such as intermittent claudication, necessitated surgical intervention; a graft replacement of the aorta was performed. Post-operatively, the patient completely recovered after 3 months of haemodialysis, and the markers that had pre-operatively suggested decreased renal bloodstream normalised with recovery of kidney function.

**Conclusions:**

To the best of our knowledge, this is the first report of severe AKI, secondary to aortic dissection, without direct renal artery obstruction, which progressed to “*temporary*” ESKD and was resolved following surgery. This case suggests that only coarctation above the renal artery branches following an aortic dissection can progress AKI to ESKD, despite the absence of radiological evidence confirming an obvious anatomical blockage. Further, indirect markers suggestive of decreased renal blood flow, such as ankle brachial indices, renal artery resistive indices, urinary excretion fractions, and hormonal changes, are useful for evaluating concomitant AKI and may indicate the need for surgical intervention after a Stanford type B aortic dissection.

## Background

**Fig. 1:**
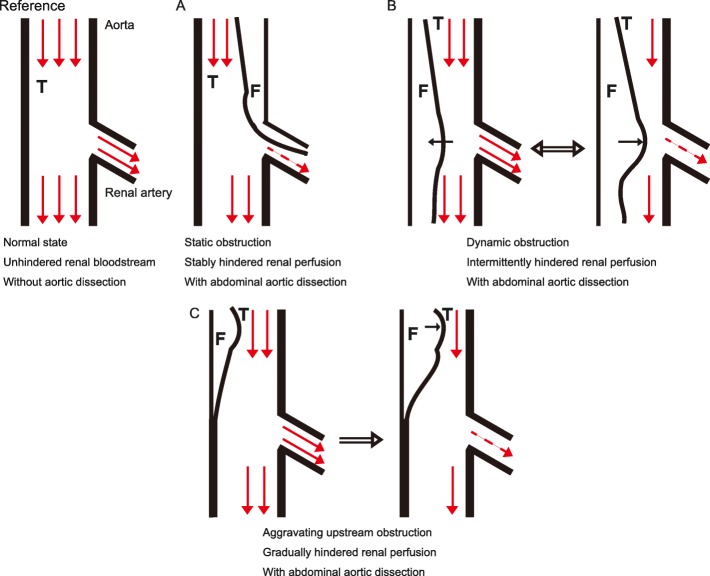
Schematic models of static, dynamic, and upstream obstructions of renal arteries and Stanford B dissection. T, true lumen; F, false lumen. Dashed and two red arrows into the renal artery indicate insufficient and sufficient blood flow, respectively. **a** Schematic model of static obstruction. Static obstruction involves renal artery stenosis or dissection into the renal artery, causing stably hindered renal perfusion, resulting in insufficient blood stream into the kidney. **b** Schematic model of dynamic obstruction. Dynamic obstruction occurs when the aortic dissection causes an intermittent blockage of renal artery perfusion, resulting in intermittently insufficient blood stream into the kidney. **c** Schematic model of upstream aortic obstruction in the patient. As time passed after admission of the patient, obstruction above the renal artery branches progressed, causing insufficient blood stream into the kidney

Acute kidney injury (AKI) is a global problem known to increase the risk of chronic kidney disease (CKD) and end-stage kidney disease (ESKD) [1–3]. Severe AKI sometimes requires renal replacement therapy (RRT) [4], but the resultant ESKD is less frequent than the resultant CKD. A meta-analysis on the long-term renal/non-renal outcomes in patients with AKI reported that the pooled incidence of CKD was 25.8 per 100 person-years, while that of ESKD was 8.6 per 100 person-years [2]. One cause of AKI is aortic dissection, reported in 4–12% of AKI cases [5]. Its pathophysiology is considered to involve either a static renal artery obstruction (Fig. 1a), such as secondary stenosis, or a dynamic obstruction, such as a flap in front of the renal artery orifices (Fig. 1b) [5, 6]. Surgical interventions for aortic dissection have rescued patients from ESKD [7–10]; these patients had static renal artery obstructions, confirmed by imaging, secondary to aortic dissections. Here, we present the first report of a patient who experienced RRT dependency for 3 months due to an aortic dissection without any imaging findings suggestive of static renal artery obstruction; his “*temporary*” ESKD unexpectedly resolved following aortic surgery. ESKD normally refers to a permanent state of dialysis dependency. Here, we use the term “*temporary*” ESKD, which refers to AKI that is severe enough to require dialysis for a period greater than 1 month but without being a permanent requirement.

## Case presentation

**Fig. 2:**
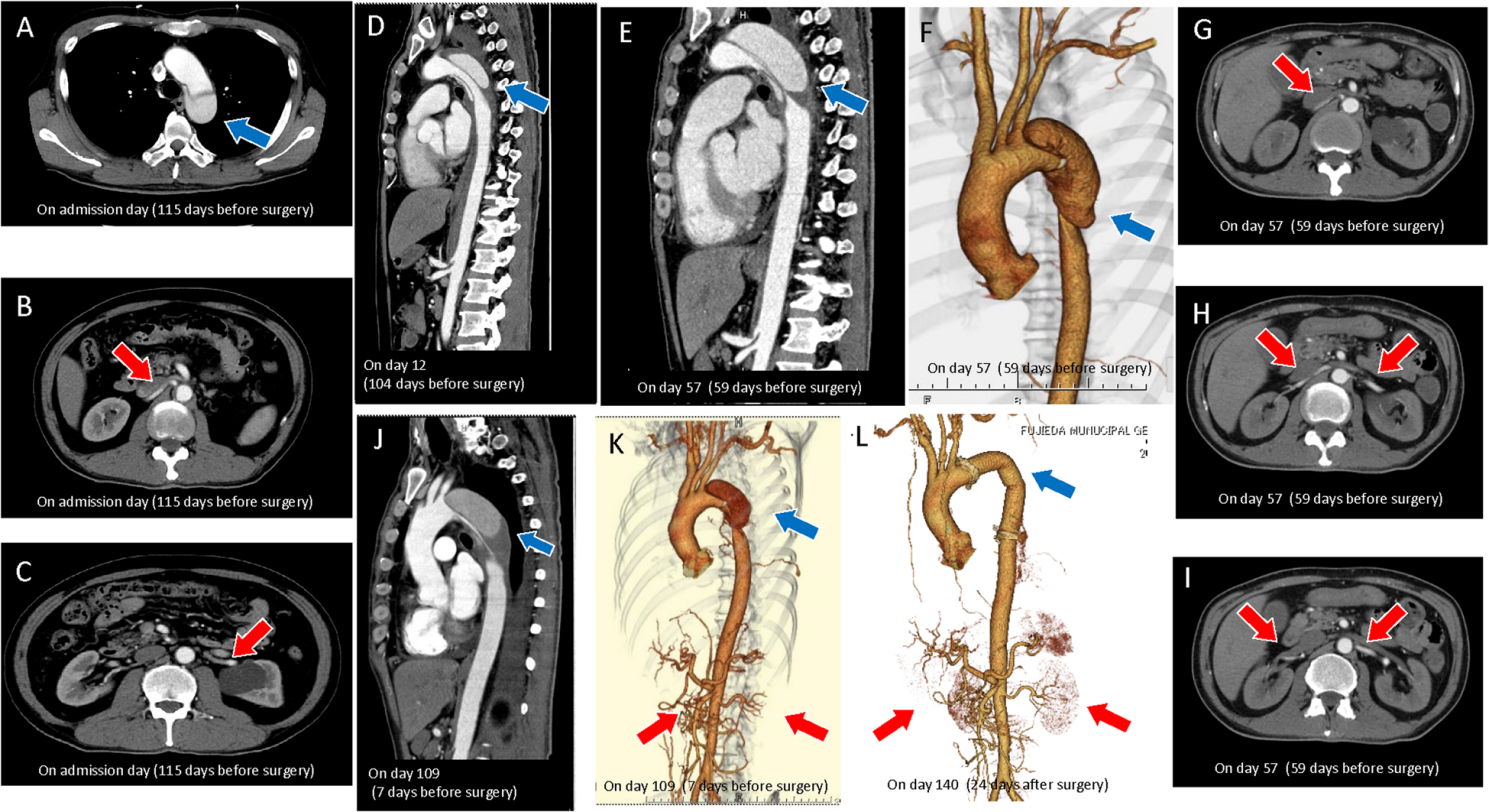
Representative aortic dissection findings upon admission and on hospital days 12, 57, 109, and 140. **a**-**c** Two-dimensional, contrast-enhanced computed tomography upon admission shows a Stanford type B aortic dissection originating from the left subclavian artery (**a**) to the abdominal aorta at the level of the renal arteries (**b**). An axial section shows no aortic dissection at the level of the renal arteries and equal contrast enhancement in both kidneys (**c**). Sagittal two-dimensional computed tomography (CT) angiography image obtained on day 12 (blue arrow) showing that the true lumen was more severely compressed by the false than that denoted on the scan taken just after admission (**d**). (**e**-**i**) CT scans of day 57. Sagittal view recorded by two-dimensional computed tomography (CT) angiography (**e**), three-dimensional CT angiography (f), and axial sections (**g**-**i**) showing the true lumen more severely compressed by the false lumen (blue arrow) than that noted on the scans taken on day 12. **j** Sagittal view recorded pre-operatively on two-dimensional CT angiography showing the true lumen more severely compressed by the false lumen (blue arrow) than that noted in the scan taken on day 57. **k** Pre-operative three-dimensional CT angiography reveals the false lumen tightly compressing the true lumen (blue arrow). Despite the compression on the thoracic part (blue arrow), on the abdominal level there is no anatomical obstruction; no static obstruction of the renal arteries is evident (red arrows). **l** After aortic graft replacement, three-dimensional CT angiography indicated that the patency of the aorta was successfully re-established (blue arrow). Changes in the morphology of the renal arteries are not apparent (red arrows). Blue arrows indicate the site of existing dissection (**a**, **d**-**f**, **j**, **k**), and the site where the graft was inserted (**l**); red arrows indicate renal arteries (**b**, **c**, **g**-**i**, **k**, **l**)

A 58-year-old man with hypertension, without renal dysfunction or family history of aortic dissection, presented with back pain and respiratory discomfort. His blood pressure was 198/110 mmHg with blood urea nitrogen (BUN) and serum creatinine (Cr) levels of 23 mg/dL and 1.8 mg/dL, respectively. The serum creatinine level a year and half before this episode was 1.12 mg/dL. The laboratory data on admission are presented in Table 1. Contrast computed tomography (CT) revealed a Stanford type B aortic dissection from the origin of the left subclavian artery to the abdominal aorta, below the divergence of the renal artery; the renal arteries were intact (Fig. 2a-c). There was little evidence of organ ischemia.

**Table 1:** Laboratory test data on admission

Laboratory data on admission (day 0)		
Parameter	Value	Reference range
Complete blood count		
Leukocytes	20,500/μL	3400–8200
Haemoglobin	13.6 g/dL	13.5–17.6
Platelets	154 × 10^3^/μL	130–370 × 10^3^
Biochemistry		
Sodium	143 mmol/L	136–147
Potassium	3.2 mmol/L	3.6–4.9
Chloride	105 mmol/L	98–108
Blood urea nitrogen	23 mg/dL	8–22
Creatinine	1.8 mg/dL	0.60–1.10
Estimated glomerular filtration rate	27 mL/min/1.73 m^2^	> 60
Aspartate aminotransferase	19 U/L	5–37
Alanine aminotransferase	15 U/L	3–35
Gamma-glutamyl transpeptidase	26 U/L	12–55
Lactate dehydrogenase	321 U/L	106–211
Creatine kinase	88 U/L	0–190
Coagulation		
PT-INR	0.89	0.85–3.00
APTT	31.1 s	25.1–36.5
D-dimer	9.1 μg/mL	< 1.0
Others		
C-reactive protein	< 0.1 mg/dL	0.00–0.20
Troponin I	Negative	

*PT-INR* Prothrombin Time and International Normalized Ratio, *APTT* Activated partial thromboplastin time

**Fig. 3:**
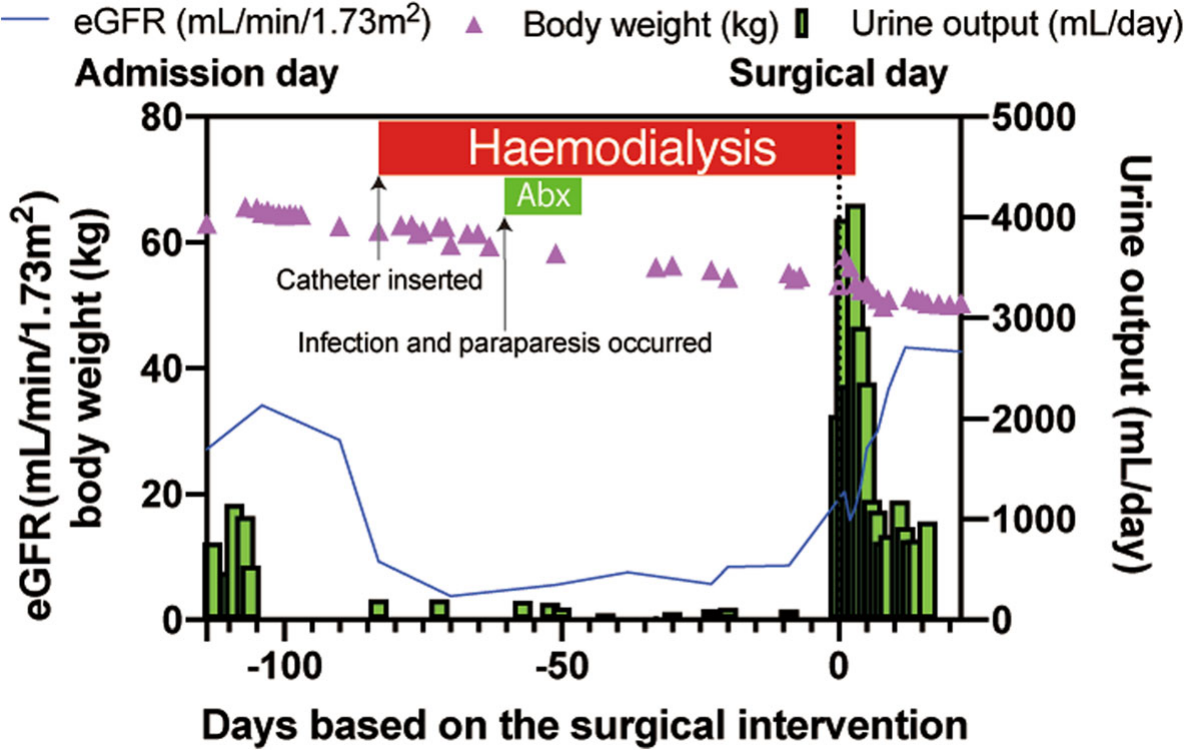
Time course of the estimated glomerular filtration rate, body weight, and urine output. After admission, the estimated glomerular filtration rate and urine volume decreased, but postoperatively, urine volume drastically increased, and the estimated glomerular filtration rate recovered. eGFR: estimated glomerular filtration rate

The patient was hospitalised to monitor and control his blood pressure and to treat the aortic dissection considering the lack of evidence of concomitant organ ischemia. Anti-hypertensives were administered, and the CT scan was repeated to check for development of ischemic complications. On day 12, the follow-up CT showed compression of the descending aorta without compression or obstruction of the renal arteries (Fig. 2d). However, 1 month after admission, his kidney function dramatically deteriorated and, laboratory data revealed severe renal dysfunction with worsening BUN (44 mg/dL) and Cr (5.6 mg/dL) levels. Abdominal ultrasonography did not suggest any structural abnormalities or chronic atrophy in the kidneys, renal artery stenosis, or decreased kidney perfusion; his resistive index (RI) values were initially 0.5–0.6. Meanwhile, hormonal data and fractional excretions of sodium (FENa) and urea nitrogen (FEUN) confirmed a prerenal pattern or decreased renal blood flow (plasma renin activity [PRA], 5 ng·mL^− 1^·hr.^− 1^; plasma aldosterone concentration [PAC], 287 pg/mL; FENa, 0.3%; FEUN, 8%). Other laboratory data did not specify the aetiology of the severe AKI apart from the prerenal AKI factors (Table 2). We continued with crystalloid fluid infusion, but the AKI was refractory. A catheter was inserted for haemodialysis, which was started 33 days after admission following acute renal failure with refractory oliguria (Fig. 3).

**Table 2:** Laboratory test data for day 32

Additional laboratory data obtained on day 32		
Parameter	Value	Reference range
Complete blood count		
Leukocytes	5600/μL	3400–8200
Haemoglobin	10.3 g/dL	13.5–17.6
Platelets	373,000/μL	130–370 × 10^3^
Biochemistry		
Sodium	136 mmol/L	136–147
Potassium	4.6 mmol/L	3.6–4.9
Chloride	102 mmol/L	98–108
Blood urea nitrogen	50 mg/Dl	8–22
Creatinine	6.5 mg/dL	0.60–1.10
Estimated glomerular filtration rate	7.9 mL/min/1.73 m^2^	> 60
Serum osmolarity	297 mOsm/kgH_2_O	270–295
Immunological assessment		
Antinuclear antibody	Negative	
Anti-DNA antibody	3 IU/mL	< 6
Anti-HCV antibody	Negative	
HBc antibody	Negative	
Glucose metabolism		
Fasting blood glucose	114 mg/dL	70–109
HbA1c	6.4%	4.6–6.2
Hormonal assessment		
Plasma renin activity	5.0 ng/mL/hr	0.2–2.7
Plasma aldosterone concentration	287 pg/mL	36–240
Urinalysis		
pH	5.5	4.5–8.0
Gravity	1.012	1.005–1.025
Red blood cell	5–9/HPF	
White blood cell	5–9/HPF	
Granular cast	Positive	
Epithelial cast	Positive	
N-acetyl-beta-D-glucosaminidase	66.9 U/L	< 11.5
Urinary α1-microglobulin	92.9 mg/L	< 8.3
U-Protein/U-Creatinine	0.30 g/gCr	< 0.15
Urinary sodium	23 mmol/l	
Urinary chloride	9 mmol/L	
Urinary urea nitrogen	213 mg/dL	
Urinary creatinine	268.6 mg/dL

On day 44, he complained of back pain, bilateral foot numbness, and paraparesis and demonstrated signs of infection (Fig. 3). Abdominal CT imaging revealed progressive thrombotic obstruction of the false lumen and compression of the true lumen in the descending thoracic aorta, while there was still no evidence of renal artery obstruction owing to the dissection (Fig. 2e-i). Thus, the paraparesis was attributed to decreased blood flow to the spinal cord. After spinal fluid drainage, the patient regained complete motor strength, and his walking improved with physiotherapy. The patient experienced intermittent claudication, and because the neurological signs and symptoms progressed owing to aortic dissection, surgery was considered. At the same time, his right and left ankle brachial indices (ABI) were 0.33 and 0.37, respectively. However, surgery was postponed because of concomitant infections, including catheter-related infection and pneumonia. The catheter-related infection was owing to methicillin-sensitive*Staphylococcus aureus* detected in the blood culture. We removed the catheter and administered ceftriaxone; however, the patient developed pneumonia in the right lobe, and we changed the antibiotic from ceftriaxone to meropenem. Thus, we administered antibiotics for 2 weeks, and confirmed a negative blood culture after treatment without any complications, such as infectious endocarditis (Fig. 3). The RI of both renal arteries decreased to 0.3–0.4; however, there was no evidence of static renal artery obstruction (Fig. 2e-i).

Four months after admission, the patient’s systemic status improved, and a prosthetic replacement of the dissected aorta was performed. Intraoperatively, we found a 3-cm tear on the distal side of the lesser curvature, near the bifurcation of the left subclavian artery. The tear was resected, and graft replacement performed (Fig. 2j–l). Surprisingly, the patient’s anuria resolved, post-operatively, despite 3 months of dialysis. Five days post-operatively, his kidney function improved with Cr and BUN levels of 1.3 and 19 mg/dL, respectively (Fig. 3). Hormonal data, FENa, and FEUN recovered as well (PRA, 0.8 ng·mL^− 1^·hr.^− 1^; PAC, 106 pg/mL; FENa, 13%, FEUN, 69%; RI of renal arteries: 0.6–0.7; ABI: 1.14–1.27). The patient successfully recovered from dialysis-dependent ESKD. Thereafter, he did not require dialysis (Cr levels, 1.3–1.5 mg/dL) and did not experience neurological after-effects.

## Discussion and conclusions

Aortic dissections result from intimal layer tears that result in blood in the media or intramural haemorrhages; conversely, a haematoma in the media leads to perforation of the intima [11]. According to the International Registry of Acute Aortic Dissection, risk factors include hypertension, pre-existing aortic aneurysm, bicuspid aortic valve, collagen diseases such as Marfan syndrome, male sex, and age >  60 years [12, 13]. Although Stanford type A dissections require emergency surgeries [14, 15], Stanford type B dissections may be managed with medication [6, 16]. However, impaired blood flow to the organs and limbs necessitates surgical intervention [17]; our patient showed progression of neurological symptoms and decreased ABI, indicative of low perfusion to the lower limbs and cardiovascular abnormality [18], and decreased renal artery RI, suggesting a further decrease in renal perfusion [19]. Generally speaking, for patients with life-threatening complications of acute type B aortic dissections emergency treatment options include open surgical aortic graft replacement; thoracic aortic stent-grafting; interventional or surgical abdominal fenestration; and catheter reperfusion or extra-anatomic surgical bypass, or both [6]. Despite its invasiveness and risk, we considered surgical graft replacement to be the most appropriate therapy, based on the patient’s age and anatomical characteristics. Therefore, after obtaining informed consent, we performed an open surgical aortic graft replacement. In another report, the doctors chose to perform thoracic endovascular aortic repair in a patient undergoing AKI owing to the dissection [20], so the surgical procedure is a matter of choice based on the characteristics of the patient and aortic dissection.

Aortic dissection often results in vascular complications, such as stroke and visceral ischemia [21]. Renal complications secondary to aortic dissection are relatively common [5], yet there are few reports on aortic dissection with concomitant AKI requiring RRT [7–10, 20, 22, 23]. The aetiologies of severe AKI due to aortic dissection in these reports were mostly limited to static obstructions (see Fig. 1a), such as stenosis or dissection. To the best of our knowledge, this is the first report of severe AKI, secondary to aortic dissection without anatomically direct renal artery obstruction, which progressed to ESKD that resolved following surgery. The accelerated AKI could have been explained by the aggravated obstruction in this patient (Fig. 1c) because of a false lumen in the descending aorta that compressed the true lumen, decreasing the downstream blood flow. Dynamic or upstream obstructions have been reported to cause malperfusions more commonly than static obstructions [5]; however, ESKD has not been previously reported.

Significant reversal of renal function in people requiring RRT is rare [24] with recovery rates of < 10% [22, 24]. The causal factors for such “*temporary*” ESKD overlap with those of AKI (e.g. acute interstitial nephritis and acute tubular necrosis) [25]. The characteristic aetiology of “*temporary*” ESKD is that the source of renal damage is mainly infectious diseases, and autoimmune diseases, which can be treatable or even curable ones [25]. Although rare, aortic dissections sometimes result in decreased renal blood flow [26]. However, patients who recover from ESKD and achieve improved kidney function have rarely had aortic dissections diagnosed as the aetiology [25], and this may be because aortic dissections have high mortality [5].

Table 3 summarises the previous reports of patients who suffered from “*temporary*” conditions necessitating dialysis for more than 1 month owing to Stanford B aortic dissection but did not require dialysis thereafter [7–10] . We have limited the cases in this table to those in whom kidney function was restored following “*temporary”* ESKD more than one-month-old because intervention against aortic dissection often complicates patients with AKI [27] and to those with Stanford B aortic dissection because Stanford A aortic dissection itself generally requires prompt operation [14, 15]. Hence, it is virtually impossible to observe patients with Stanford A aortic dissection without performing any interventions for more than a month. Although the risk factors of post-surgical AKI following aortic dissection are identified [27], those of pre-surgical AKI, especially AKI necessitating RRT following aortic dissection are unknown owing to the limited number of reports [7–10, 20, 22, 23], Based on the data in Table 3, all patients had a history of hypertension in common, which is a risk factor for aortic aneurysm [12, 13]; thus, hypertension may be a risk factor for pre-surgical AKI requiring RRT owing to Stanford type B aortic dissection. We also observed from Table 3 that three out of the five patients underwent percutaneous intervention instead of surgical procedure, which could be attributed to the patients’ possible intolerability to surgery or characteristics of aortic dissection [7–9]. Surprisingly, in one of the 5 reports, the patient was relieved of dialysis without any intervention specific to the dissection [10]; however, this scenario seems rare. This hypothesis was supported by the fact that another ESKD patient following Stanford B aortic dissection who did not undergo surgical or radiological treatment, continued to be on permanent dialysis [22].

**Table 3:** Characteristics of patients rescued from ESKD lasting longer than 1 month in previous reports and this report

Authors	Year of report	Sex	Age	Kidney type	Pre-existing conditions	Mechanism of AKI	Dialysis-dependent period	Intervention
Lacombe P et al [7]	1992	Male	45	Naïve	Hypertension	Static obstruction to left renal artery	6 weeks	Percutaneous catheterization of left renal artery
Kammerl MC et al [8]	1999	Male	47	Naïve	Hypertension, nephrotic syndrome	Static obstruction to left renal artery	2.5 months	Percutaneous catheterization of both aorta and left renal artery
Weiss AS et al [9]	2004	Male	69	Naïve	Hypertension	Static obstruction to both renal arteries	3 months	Percutaneous catheterization of left renal artery
Dujardin A et al [10]	2017	Male	63	Transplanted	Hypertension, renal transplantation	Static obstruction to right femoral artery	8 months	Medications for kidney transplantation
This report	2019	Male	58	Naïve	Hypertension	Only dynamic obstruction radiologically confirmed	3 months	Surgical graft replacement

*ESKD* End-stage kidney disease

As in this report, if a patient does not have evidence of a static renal obstruction following an aortic dissection that causes severe AKI, uncovering the association is very difficult. Despite the absence of radiological evidence, the aortic dissection was the primary cause, because the surgical intervention put an end to the RRT dependency of the patient. Of course, other factors that cause AKI could have exacerbated the AKI. For instance, recurrent infections can worsen AKI, since infection is a major cause of AKI [28]. In fact, after the admission of the patient, the patient experienced several severe infections, such as vascular catheter-related infections or pneumonia. However, the association of AKI and infection in our case is weak because the infectious diseases originated from a 2-week-old catheter while undergoing haemodialysis; hence, the patient was already dialysis-dependent at the time of the first infection.

After diagnosing the aortic dissection, we provided conservative medical treatment with careful monitoring for possible complications of Stanford type B dissection [6]. Although the patient’s kidney function deteriorated after admission, no radiological evidence of its association with the dissection was initially available. Initial differential diagnoses included infections, anatomical renal artery constriction, and renal embolism due to the dissection. The former probably affected renal function deterioration partially, but the CT and ultrasonography images supported the absence of the latter two. We were at a loss as to what caused the severe AKI initially; surgical treatment became absolutely necessary because of the vertebral infarction that occurred a few months after admission—an obvious complication of aortic dissection. However, in retrospect, indirect signs of renal ischemia were present, such as the decreased renal artery RI, which suggested severely low renal perfusion [19, 29]. Our evaluation method was in accordance with the report by Crawford et al. who described the usefulness of renal artery Doppler ultrasonography for evaluating renal ischemia due to aortic dissection [5]. Additionally, low FENa and FEUN values strongly suggested prerenal AKI [30]; therefore, the low FENa and FEUN values and the ineffectiveness of the crystalloid fluid infusion suggested decreased blood flow due to the dissection. Furthermore, the high PRA and PRA/PAC values were suggestive of decreased renal blood supply [31]. Post-operatively, the PRA/PAC ratio, FENa, FEUN, renal artery RIs, and ABI normalised. When an aortic dissection does not extend into the renal arteries, deciding on the appropriate stage for surgical treatment is difficult. In such cases, these parameters may be promising indicators of the need for surgery.

In summary, the patient developed “*temporary”* ESKD owing to severe prerenal AKI caused by aortic dissection, notably without any anatomically direct obstruction of the renal arteries and did not require dialysis after the surgery. This case also highlights the usefulness of renal Doppler ultrasonography, urinary excretion fractions, and hormonal changes for evaluating renal blood perfusion, even in the absence of radiological signs of anatomical renal artery obstruction due to aortic dissection.

## Data Availability

The dataset supporting the conclusions of this article is included within the article.

## Abbreviations

ABI: Ankle brachial indices
AKI: Acute kidney injury
BUN: Blood urea nitrogen
Cr: Serum creatinine
CT: Computed tomography
ESKD: End-stage kidney disease
FENa: Fractional excretion of sodium
FEUN: Fractional excretion of urea nitrogen
PAC: Plasma aldosterone concentration
PRA: Plasma renin activity
RI: Resistive index
RRT: Renal replacement therapy

## References

[1] Coca SG (2010). Acute kidney injury in elderly persons. Am J Kidney Dis.

[2] Coca SG, Singanamala S, Parikh CR (2012). Chronic kidney disease after acute kidney injury: a systematic review and meta-analysis. Kidney Int.

[3] Basile DP, Bonventre JV, Mehta R, Nangaku M, Unwin R, Rosner MH (2016). ADQI XIII work group progression after AKI: understanding maladaptive repair processes to predict and identify therapeutic treatments. J Am Soc Nephrol.

[4] Iwagami M, Yasunaga H, Noiri E, Horiguchi H, Fushimi K, Matsubara T (2015). Current state of continuous renal replacement therapy for acute kidney injury in Japanese intensive care units in 2011: analysis of a national administrative database. Nephrol Dial Transplant.

[5] Crawford TC, Beaulieu RJ, Ehlert BA, Ratchford EV, Black JH (2016). Malperfusion syndromes in aortic dissections. Vasc Med.

[6] Uchida N, Shibamura H, Katayama A, Aishin K, Sutoh M, Kuraoka M (2009). Surgical strategies for organ malperfusions in acute type B aortic dissection. Interact Cardiovasc Thorac Surg.

[7] Lacombe P, Mulot R, Labedan F, Jondeau G, Barré O, Chagnon S (1992). Percutaneous recanalization of a renal artery in aortic dissection. Radiology..

[8] Kammerl MC, Manke C, Feuerbach S, Reber D, Aebert H, Birnbaum D (1999). Cure of apparent end-stage renal disease in a patient with dissecting aneurysm of the aorta using a percutaneous interventional approach. Nephrol Dial Transplant.

[9] Weiss AS, Ludkowski M, Parikh CR (2004). Reversal of end-stage renal disease after aortic dissection using renal artery stent: a case report. BMC Nephrol.

[10] Dujardin A, Le Fur A, Cantarovich D (2017). Aortic dissection and severe renal failure 6 years after kidney transplantation. Transplant Direct.

[11] Larson EW, Edwards WD (1984). Risk factors for aortic dissection: a necropsy study of 161 cases. Am J Cardiol.

[12] Hagan PG, Nienaber CA, Isselbacher EM, Bruckman D, Karavite DJ, Russman PL (2000). The international registry of acute aortic dissection (IRAD): new insights into an old disease. JAMA..

[13] Januzzi JL, Isselbacher EM, Fattori R, Bruckman D, Karavite DJ, Russman PL (2000). Characterizing the young patient with aortic dissection: results from the international registry of aortic dissection (IRAD). J Am Coll Cardiol.

[14] Pacini D, Leone A, Belotti LM, Fortuna D, Gabbieri D, Zussa C (2013). Acute type a aortic dissection: significance of multiorgan malperfusion. Eur J Cardiothorac Surg.

[15] Di Eusanio M, Trimarchi S, Patel HJ, Hutchison S, Suzuki T, Peterson MD (2013). Clinical presentation, management, and short-term outcome of patients with type a acute dissection complicated by mesenteric malperfusion: observations from the international registry of acute aortic dissection. J Thorac Cardiovasc Surg.

[16] Tsai TT, Nienaber CA, Eagle KA (2005). Acute aortic syndromes. Circulation..

[17] Khan IA, Nair CK (2002). Clinical, diagnostic, and management perspectives of aortic dissection. Chest..

[18] Criqui MH, Aboyans V, Allison MA, Denenberg JO, Forbang N, McDermott MM (2016). Peripheral artery disease and aortic disease. Glob Heart.

[19] Schwerk WB, Restrepo IK, Stellwaag M, Klose KJ, Schade-Brittinger C (1994). Renal artery stenosis: grading with image-directed Doppler US evaluation of renal resistive index. Radiology..

[20] Li L, Zhuang S, Qi S, Cui J, Zhou J, Zhu H (2013). Acute thoracic aortic dissection (Stanford type B) complicated with acute renal failure. Case Rep Vasc Med.

[21] Cambria RP, Brewster DC, Gertler J, Moncure AC, Gusberg R, Tilson MD (1988). Vascular complications associated with spontaneous aortic dissection. J Vasc Surg.

[22] Brooke V, Goswami S, Mohanty A, Kasi PM (2012). Aortic dissection and renal failure in a patient with severe hypothyroidism. Case Rep Med.

[23] Galabada DP, Nazar AL (2014). Unusual presentation of aortic dissection: post-coital acute paraplegia with renal failure. Saudi J Kidney Dis Transpl.

[24] Mohan S, Huff E, Wish J, Lilly M, Chen SC, McClellan WM, Fistula first breakthrough initiative data committee (2013). Recovery of renal function among ESRD patients in the US Medicare program. PloS One.

[25] Macdonald JA, McDonald SP, Hawley CM, Rosman J, Brown F, Wiggins KJ (2009). Recovery of renal function in end-stage renal failure--comparison between peritoneal dialysis and haemodialysis. Nephrol Dial Transplant.

[26] Siegelman SS, Sprayregen S, Strasberg Z, Attai LA, Robinson G (1970). Aortic dissection and the left renal artery. Radiology..

[27] Zhang Z, Ni H (2015). Normalized lactate load is associated with development of acute kidney injury in patients who underwent cardiopulmonary bypass surgery. PLoS One.

[28] Kopolovic I, Simmonds K, Duggan S, Ewanchuk M, Stollery DE, Bagshaw SM (2013). Risk factors and outcomes associated with acute kidney injury following ruptured abdominal aortic aneurysm. BMC Nephrol.

[29] Riehl J, Schmitt H, Bongartz D, Bergmann D, Sieberth HG (1997). Renal artery stenosis: evaluation with colour duplex ultrasonography. Nephrol Dialy Transplant.

[30] Pepin MN, Bouchard J, Legault L, Ethier J (2007). Diagnostic performance of fractional excretion of urea and fractional excretion of sodium in the evaluations of patients with acute kidney injury with or without diuretic treatment. Am J Kidney Dis.

[31] Kotliar C, Inserra F, Forcada P, Cavanagh E, Obregon S, Navari C (2010). Are plasma renin activity and aldosterone levels useful as a screening test to differentiate between unilateral and bilateral renal artery stenosis in hypertensive patients?. J Hypertens.

